# Monitoring Virgin Olive Oil Shelf-Life by Fluorescence Spectroscopy and Sensory Characteristics: A Multidimensional Study Carried Out under Simulated Market Conditions

**DOI:** 10.3390/foods9121846

**Published:** 2020-12-11

**Authors:** Ana Lobo-Prieto, Noelia Tena, Ramón Aparicio-Ruiz, Diego L. García-González, Ewa Sikorska

**Affiliations:** 1 Instituto de la Grasa (CSIC), Campus Universidad Pablo de Olavide-Edificio 46, Ctra. de Utrera, Km. 1, 41013 Sevilla, Spain; ana.lobo@ig.csic.es; 2 Department of Analytical Chemistry, Universidad de Sevilla, C/Prof. García González 2, 41012 Sevilla, Spain; ntena@us.es (N.T.); aparicioruiz@us.es (R.A.-R.); 3 Institute of Quality Science, The Poznan University of Economics and Business, al. Niepodleglosci 10, 61-875 Poznan, Poland; ewa.sikorska@ue.poznan.pl

**Keywords:** fluorescence spectroscopy, virgin olive oil, shelf-life, PARAFAC, sensory assessment, quality

## Abstract

The control of virgin olive oil (VOO) freshness requires new tools that reflect the diverse chemical changes that take place during the market period. Fluorescence spectroscopy is one of the techniques that has been suggested for controlling virgin olive oil (VOO) freshness during its shelf-life. However, a complete interpretation of fluorescence spectra requires analyzing multiple parameters (chemical, physical–chemical, and sensory) to evaluate the pace of fluorescence spectral changes under moderate conditions with respect to other changes impacting on VOO quality. In this work, four VOOs were analyzed every month with excitation–emission fluorescence spectra. The same samples were characterized with the concentration of fluorophores (phenols, tocopherols, chlorophyll pigments), physical–chemical parameters (peroxide value, K_232_, K_270_, free acidity), and sensory attributes (medians of defects and of the fruity attribute). From the six components extracted with parallel factor analysis (PARAFAC), two components were assigned to chlorophyll pigments and those assigned to tocopherols, phenols, and oxidation products were selected for their ability to discriminate between fresh and aged oils. Thus, the component assigned to oxidation products correlated with K_270_ in the range 0.80–0.93, while the component assigned to tocopherols–phenols correlated with the fruity attribute in the range 0.52–0.90. The sensory analysis of the samples revealed that the changes of these PARAFAC components occurred at the same time as, or even before, the changes of the sensory characteristics.

## 1. Introduction

The healthy and organoleptic properties of virgin olive oil (VOO) make this product highly valued by consumers. Furthermore, due to the current preference of consumers for less-processed products, VOO is already consumed in greater quantity and for more countries than ten years ago [1]. Since VOO is only produced during a few months per year, it must be stored and carefully handled to guarantee the supply during the entire year. Several studies have focused on tracking the chemical alteration of VOO during its storage under different conditions [2,3,4]. The oxidation process causes the loss of its antioxidant compounds and the reduction of its sensory and healthy properties. These changes may even lead to a downgrading of the category (e.g., from “extra virgin olive oil” to “virgin olive oil”), with the resulting reduction of the product value and consumer acceptability [5]. For that reason, quality control of VOO during its shelf-life is a current concern in the olive oil sector. The regulatory bodies have established several individual parameters to determine the quality and oxidation state of VOO. However, the complexity of the degradation process, where many parameters are involved and can influenced by each other, make necessary the development of new analytical tools that are able to assess the quality state of VOO since a multiparametric perspective.

Light and temperature, even under moderate conditions, have a great influence on the degradation of VOO [6,7,8,9,10]. Several studies have been focused on the effect of light and temperature upon VOO shelf-life [11,12,13]. These studies have highlighted the strong effect of light on the VOO oxidation stability; nevertheless, the response of VOO to moderate light and temperature is highly conditioned by the chemical composition of the oil [14]. During its sale and distribution, VOO stability can be considerably affected in different manners by these and others variables, which may lead to some disparities between the results of control testing (quality parameters, Rancimat method, etc.) and “the best before date” declared on the label. This problem has caused a deep concern in the regulatory and control bodies, which have established some guidelines for an optimum storage of olive oil [15,16] in order to prevent a rapid degradation of the oils. However, they do not establish a maximum period of storage or specify how VOO degradation could be controlled during the storage. Thus, currently, the analytical tools to ensure the VOO freshness and quality during the storage are not clear enough in their interpretation. The lack of harmonized analytical tools for this purpose means that most producers define the “shelf-life” of each VOO batch following their own criteria. In consequence, some discrepancies are sometimes found between the actual quality of the product in a supermarket and the quality declared on the label and expected by consumers when they purchase it.

Although there are several methods available to estimate the VOO stability, such as the oil stability index or active oxygen method, they use experimental conditions that are different from those found in actual storage (e.g., a temperature of 100 °C or more is applied). These differences in conditions modify the kinetics of the oxidation process and its effect upon VOO when it is stored under moderate conditions [14,17]. The control of the degradation process involves the monitoring of many parameters (peroxide value, free acidity, ultraviolet absorbance, organoleptic assessment, phenol content, etc.) with different time-trends in the course of the storage and informing about a particular aspect of quality, which makes it difficult to interpret quality with an overall perspective.

The need for controlling a high number of parameters during the different steps of the food chain, including storage, turns spectroscopy into an adequate technique capable of providing global information of the quality state of foods. Spectroscopic techniques have been extensively applied in food analysis since they allow a rapid and efficient measurement of a large variety of chemical parameters in food matrices [18]. Due to the usefulness and simplicity of measurements with these techniques, they are presented as an effective alternative to the classical analytical methods [19]. Currently, their applications in the analysis of different edible vegetable oils is rapidly growing.

Particularly, total luminescence spectroscopy has been implemented in food analysis and it permits the characterization of samples regarding different quality and authenticity issues [20]. Thus, it is presented as a useful and accurate technique to get information about the fluorescence compounds in vegetable oils, this technique being able to detect lower concentrations than absorption spectroscopy [21]. Total luminescence spectroscopy provides the total intensity profile associated with the fluorescent compounds present in a sample in a determined excitation and emission range of wavelengths. The obtained excitation–emission matrix (EEM) is a three-dimensional spectrum or contour map, and contains signals from all fluorophores that are present in the sample. The analysis of the EEMs with multivariate methods allows information to be extracted about the different fluorescent compounds found in a food sample simultaneously. This characteristic makes total luminescence spectroscopy an adequate analytical technique to study virgin olive oil (VOO), which is a complex food system that contains several fluorescent compounds, such as phenols, tocopherols, and pheophytins.

Several authors have proposed using fluorescence spectroscopy to characterize VOO [22,23,24,25,26]. Other authors have applied this technique to detect frauds such as blends of olive oils with other vegetable oils [27,28]. Furthermore, fluorescence spectroscopy has also been proposed to discriminate oils with different geographical provenances [29]. The tracking of the different fluorophores during the VOO storage provides global information about the chemical changes taking place, and consequently, provides knowledge of how the oxidation process progresses depending on the VOO chemical composition and the storage conditions.

This study proposes the total luminescence spectroscopy combined with (parallel factor) PARAFAC analysis as an appropriate technique, which is able to monitor the changes of VOO produced during the storage under moderate conditions from a multidimensional perspective. Four monovarietal VOOs from three different cultivars were stored during 21 months under conditions close to the real ones, in order to study their fluorescence characteristics, and, at the same time, the chemical quality parameters and sensory attributes. The objective of this study was to verify if the results obtained from fluorescence spectroscopy and chemometrics could provide real information of the changes occurring in VOO during its storage, and the ability of this method to distinguish between fresh and aged oils. Furthermore, due to the relevance of the sensory quality of VOO, the results obtained by an accredited panel are included to have complete information about the quality of the oils.

## 2. Materials and Methods

### 2.1. Samples

Four virgin olive oils (VOOs) from Picual, Hojiblanca, and Arbequina (2 oils) cultivars were used in this study. These three cultivars were selected because of their distribution and for being predominant in a particular region. The four VOOs were directly provided by Spanish producers and they were taken from the vertical centrifuge at the oil mill, in order to guarantee the freshness of the oils. Subsequently, the VOOs were filtered to remove moisture, and bottled. The filtration in the laboratory was carried out in the dark to avoid photooxidation, using folded filter paper (filter paper 600, Dorsan Living Filtration, Barcelona, Spain). After the filtration, the samples were randomly subjected to moisture analysis according to ISO 662 to check the filtration efficiency. Three randomly selected portions of each oils were analyzed in duplicate and the moisture contents were below 0.1% *m*/*m* in all cases. The samples were named as follows: VOO1, Hojiblanca; VOO2, Arbequina-A; VOO3, Picual; VOO4, Arbequina-B. 

### 2.2. Storage Experiment

VOOs were stored for 21 months in a compartment where the conditions that are given in a supermarket were simulated. Each VOO was packaged in 22 transparent PET (polyethylene terephthalate) bottles of 500 mL (one per month of storage plus one for the fresh sample), and they were hermetically sealed. The bottles were kept under a light intensity of ≈1000 lx in 12 h light/dark cycles, while the temperature and humidity were controlled. In this storage experiment, the temperature, daily controlled, varied between 16.3 and 29.7 °C, and the humidity varied between 21% and 70%. During the storage experiment, one bottle was opened and analyzed every month. The oil remained in the bottle after the analyses were discarded. Therefore, only oils from a freshly opened bottle were used for the analyses.

### 2.3. Quality Parameters

The quality parameters were analyzed in the fresh samples (time zero) to determine the VOO category before starting the storage. During the storage experiment, they were also analyzed each month in order to monitor their changes. These parameters were the peroxide value (PV), free fatty acid content (FFA), and extinction coefficients from ultra-violet absorbance (K_270_ and K_232_), which were measured by applying the International Olive Council methods [30,31,32].

### 2.4. Sensory Assessment

The sensory characteristics of the VOO samples were determined by the panel of Instituto de la Grasa [33] applying the standard COI/T.20/Doc. No 15/Rev.10 2018 [34]. The panelists evaluated the median of the fruity attribute (Mf) and defect (Md) for the four VOOs subjected to the storage experiment. The sensory assessment results were generated every month. Thus, it provided chronological information about the sensory characteristics’ changes of the oils during the storage period, which made it possible to identify changes in the quality category of the oils and in their sensory characteristics.

### 2.5. Phenol Content

The phenol composition were determined by applying the method described by Mateos et al. [35], slightly modified by Aparicio–Ruiz et al. [36]. An amount of 2.5 g of the sample was solved in 6 mL of hexane, and *p*-hydroxyphenylacetic (0.12 mg/mL) and *o*-coumaric (0.01 mg/mL) were added as internal standards. The isolation of the phenolic fraction was carried out with methanol by solid phase extraction using diol-bonded phase cartridges. After that, the concentrated phenolic fraction was injected in the HPLC system (Agilent Technologies 1200, Waghaeusel–Wiesental, Germany), equipped with a diode array detector. The column was a Lichrospher 100RP-18 column (4.0 i.d. × 250 mm; 5 µm, particle size) (Darmstadt, Germany) kept at 30 °C. The flow rate of 1.0 mL/min was used and the gradient elution was performed using a mixture of water/ortho-phosphoric acid (99.5:0.5 *v*/*v*) (solvent A) and methanol/acetonitrile (50:50 *v*/*v*) (solvent B). The change in solvent gradient was programed as follows: From 95% (A) and 5% (B) to 70% (A) and 30% (B) in 25 min; 65% (A) and 35% (B) in 10 min; 60% (A) and 40% (B) in 5 min; 30% (A) and 70% (B) in 10 min and 100% (B) in 5 min, followed by 5 min of maintenance. The chromatographic signals were obtained at 235, 280, and 335 nm. Figure S1 shows an example of a chromatogram obtained in the analysis and Table S2 shows the phenolic compounds identified in the VOO samples. The quantification of the phenols was carried out following the procedure described by Mateos et al. [35]. Quantification of phenols, lignans, and cinnamic acid was carried out at 280 nm using *p*-hydroxyphenylacetic acid as internal standard, whereas the quantification of flavones was at 335 nm using *o*-coumaric acid as internal standard.

### 2.6. α-Tocopherol Content

The method ISO 9936:2016 [37] was applied for the determination of α-tocopherol. The sample (0.1 g) was dissolved with 10 mL of hexane. From this solution, 20 µL was injected into the HPLC Agilent Technologies 1200 (Waghaeusel–Wiesental, Germany), equipped with a fluorescence detector. The column used was a silica gel column Superspher^®^RP-18 (4 i.d. × 250 mm length, 5µm particle size) purchased from Merck (Darmstadt, Germany). The identification of α-tocopherol was carried out with λ_ex_ = 290 nm and λ_em_ = 330 nm. Figure S2 shows an example of a chromatogram obtained in this analysis. The quantification of α-tocopherol was carried out by calibration curve of a stock solution of α-tocopherol (Sigma–Aldrich–Fluka, Darmstadt, Germany). The preparation of the stock solution and the development of its calibration curve was carried out following ISO 9936:2016 [37]. The real concentration of the stock solution was determined by its maximum absorbance in a wavelength range between 270 and 310 nm using a UV VIS spectrometer Thermo Scientific GENESYS 10s (Waltham, MA, USA) and 10-mm path length cell, Hellma Analytics (Müllheim, Germany).

### 2.7. Pigment Analysis

The determination of the degradation products of chlorophyll a, such as pheophytin a and pyropheophytin a, were measured using the method ISO 29841:2012 [37]. The fraction of chlorophyll pigment was extracted by solid phase extraction using silica cartridge 1000 mg/6 mL, 55 µm, 700 nm (Supelco, Bellefonte, PA, USA). The analysis of pigments was carried out using an HPLC system LaChrom Elite de Hitachi (Tokyo, Japan) with a diode array detector. The column used was a Lichrospher RP18 HPLC column, 250 mm length, 4.0 mm internal diameter, filled with reversed-phase particles size 5 µm (Merck, Darmstadt, Germany). The peak identification was carried out using the standard of pheophytin a and pyropheophytin a, which were obtained from a dissolution of ethyl ether and chlorophyll a, from spinach (Sigma–Aldrich, Darmstadt, Germany), following the procedure explained by Sierves and Hynninen [38] in the case of pheophytin a, and Schwartz et al. [39] in the case of pyropheophytin a. Figure S3 shows an example of a chromatogram of the degradation products of chlorophyll a obtained in the analysis. 

### 2.8. Fluorescence Measurements

The fluorescence spectra were obtained with an AqualogTM (Horiba, Montpellier, France) spectrofluorometer. A xenon lamp was used as an excitation source. Before the measurements, the instrument performance was checked using a standard procedure. The excitation and emission slit widths were 5 nm. The gain of a charge-coupled device (CCD) detector was set to a low range. The corrected three-dimensional spectra were obtained by measuring the emission spectra from 250 to 830 nm with an average increment of 4.66 nm repeatedly, at excitation wavelengths from 240 to 800 nm, spaced by 5 nm intervals. Right-angle geometry was used for analyzing the oil samples diluted in n-hexane (3% *v*/*v*) in a 10-mm fused-quartz cuvette. This low concentration was chosen to avoid spectral distortions. Additionally, the inner filter effect was corrected based on the simultaneous absorbance measurements, using AqualogTM built-in software.

### 2.9. Statistical Analysis

The excitation–emission matrices in the excitation range of 280–800 nm and emission range of 300–830 nm (EEMs) were used for the statistical analysis. The EEMs of 22 samples per each of the four oils were arranged in a three-dimensional structure with a size of 88 × 114 × 105 (number of samples x number of emission wavelengths x number of excitation wavelengths). The entire data set was analyzed using PARAFAC, which is able to break down the EEMs into the contributions of the individual fluorescent components. The Rayleigh scattering bands were removed with a Rayleigh-masking algorithm. Core consistency diagnostics (CONCORDIA) and the explained variance were used to find the optimal number of components in the PARAFAC model [40,41]. The PARAFAC analysis was carried out with SOLO v.8.7.1 software (Eigenvector Research Inc., Wenatchee, WA, USA).

Principal component analysis (PCA) was performed with the chemical parameters (quality parameters, phenols, and α-tocopherol content) analyzed in the stored samples and the fluorescence components extracted by PARAFAC analysis. The loading plot and the scores plot were studied in order to identify the relationship between the PARAFAC components and the chemical parameters, and to characterize the stored VOOs. 

Stepwise linear discriminant analysis (SLDA) was performed in fresh (0–5 months) and aged (16–21 months) samples using the fluorescence components obtained by PARAFAC, in order to identify the PARAFAC components that were able to discriminate between these two kinds of samples. Significance discrimination was accepted when *p* < 0.05.

The multivariate analyses were carried out using the STATISTICA 8 package (Statsoft, Tulsa, OK, USA).

## 3. Results and Discussion

### 3.1. Physical–Chemical Characterization

Peroxide value, free acidity (free fatty acids or FFA), extinction coefficients (K_270_ and K_232_), and the total concentration of phenols, α-tocopherol, and pigments derived from chlorophyll a (pheophytin a and pyropheophytin a) were analyzed in the four fresh VOOs previous to the storage (“time zero”) in order to characterize the VOOs at the moment of bottling. Table 1 shows the results for peroxide value, free acidity, and K_270_ and K_232_ for each VOO during the storage experiment. Furthermore, Table 2 shows the concentrations of phenols, α-tocopherol, pheophytin a, and pyropheophytin a for each VOO during the entire experiment. The values of peroxide value, FFA, K_270_, and K_232_ in the fresh samples (“time zero”) revealed that the four VOOs belonged to the “extra virgin olive oil” category, according to the European Commission (EC) regulation [15]. VOO2 showed the highest value of peroxide value, K_270_, and K_232_, which pointed out a certain degree of alteration despite the fact that all the VOOs were fresh and they were directly taken from the vertical centrifuge.

Regarding the concentration of phenols, VOO3 and VOO4 were characterized with the highest concentrations, 564.82 and 451.25 mg/kg, respectively. VOO1 and VOO2 showed lower concentrations of total phenols (Table 2), 246.71 and 338.90 mg/kg respectively. The method used for this determination was successfully used in previous works [35,36]. This method allowed for a good separation of several kinds of phenols in a single chromatographic run. 

The initial concentration of α-tocopherol was similar between VOO2 and VOO3, with a value of 272.28 and 256.91 mg/kg. The other two VOOs showed a lower concentration, 212.62 in VOO1 and 192.94 mg/kg in VOO4 (Table 2). The study of minor compounds, such as phenolic and tocopherols compounds, can provide information about how stable the oil is under oxidation, since they interrupt the propagation chain of lipid oxidation [42,43,44].

The tracking of pheophytin a and pyropheophytin a concentrations in VOO over time have been used in the monitoring of its stability and its loss of freshness [45]. The highest concentration of pheophytin a in the fresh samples (Table 2) was found in VOO3 with a value of 23.43 mg/kg. The highest concentration of pyropheophytin a in the fresh oils was found in VOO4 with a value of 0.11 mg/kg.

As soon as the storage experiment started, all the quality parameters evolved immediately (Table 1 and Table 2). The quality indexes (peroxide value, free acidity, K_270_ and K_232_) showed their maximum values in the last month of storage, although they were within the “extra virgin olive oil” category according to the limits stated in European regulation [15], except for K_270_ (Table 1). This parameter surpassed the limit established for the “extra virgin olive oil” category in the first months of storage (2–4 month) for the four VOOs. VOO1 and VOO3 showed the highest K_270_ values, while VOO2 and VOO4 showed the highest value of K_232_. The rest of the parameters, phenols, α-tocopherol, and pheophytin a, decreased their concentration from the beginning of the storage (Table 2). Pyropheophytin a concentration also increased from the beginning of the storage, but it later decreased until the end of the experiment. In this case, these compounds had fluorescence properties and their changes were reflected in the fluorescence spectra.

### 3.2. Changes of Fluorescence Excitation–Emission Matrices during the Storage

The time-trend of the main fluorescence compounds present in the stored VOOs were studied by excitation–emission fluorescence spectroscopy. All these data were studied simultaneously during the whole storage experiment, in order to obtain a multiparametric perspective of the degradation process of VOO under moderate conditions. 

**Figure 1:**
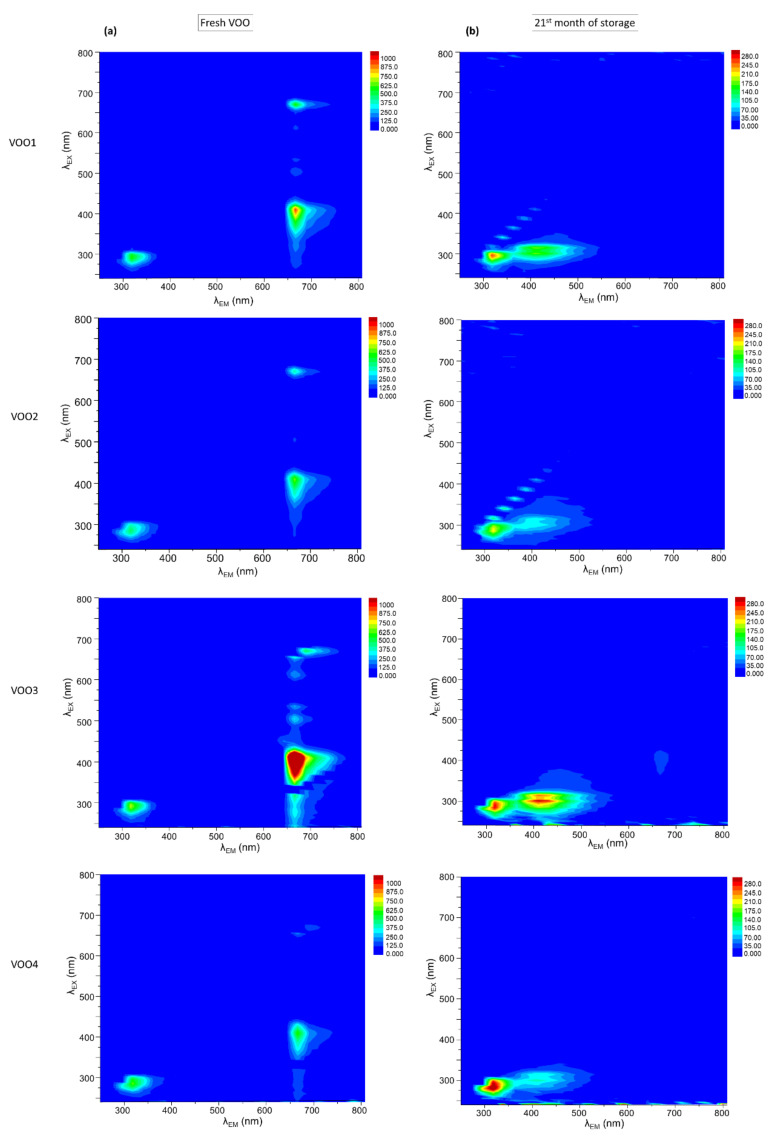
Contour maps of the excitation–emission matrices (EEMs) of the stored VOOs in two moments of their storage under moderate conditions: (**a**) Before starting the storage (fresh sample) and (**b**) at the end of the storage (twenty-first month of storage).

Figure 1a shows the contour maps of excitation–emission matrices (EEMs) of the four stored VOOs before starting the storage (fresh oils). The EEMs exhibited general features as other authors reported in previous works [4,46]. Thus, their EEMs showed two groups of bands observed in all the oils (Figure 1a). A group of bands was found in the emission wavelengths range of 600–700 nm. The most intense band in this group was found at the excitation/emission maximum (λ_ex_/λ_em_) of 408/678 nm in all the samples studied. According to previous works, this band is associated with the presence of chlorophyll pigments, mainly pheophytin a [2,4,19,47]. The second group of bands was identified at 250–350 nm of emission wavelengths, the excitation/emission maximum (λ_ex_/λ_em_) being found at 293/322 nm in all the oils. This band corresponded simultaneously to tocopherols and phenols, as it was extensively reported in previous works [19,21,24,26].

**Figure 2:**
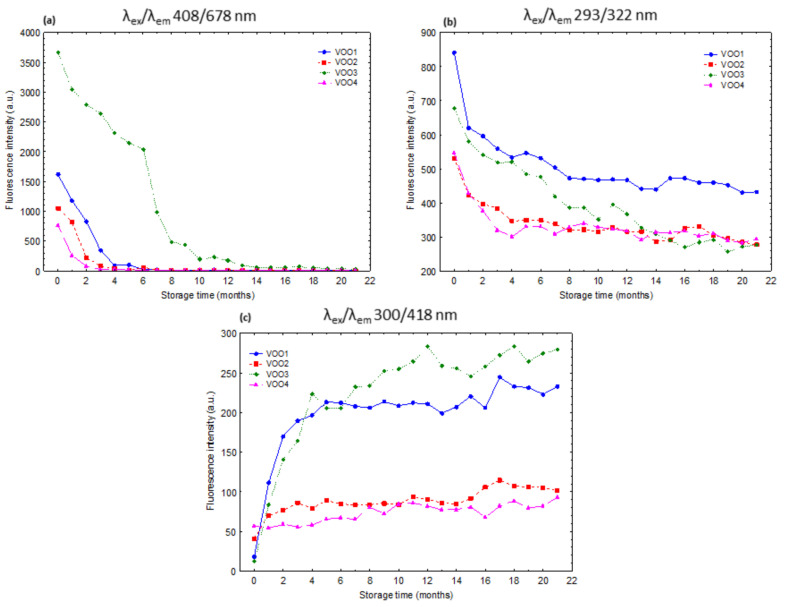
Time-trend of the fluorescence intensity of the bands found at the excitation/emission maxima of (**a**) 408/678, (**b**) 293/322, and (**c**) 300/418 nm for the virgin olive oils during the storage under moderate conditions.

Figure 1b shows the changes of the contour maps of the EEMs of the VOOs during the storage under moderate conditions. Particularly, this figure shows the EEMs at the last months of the storage (21 months). The two groups of bands associated with the fresh oils (Figure 1a) decreased progressively during the storage. Figure 2a,b display the time-trends of the bands associated with pigments (λ_ex_/λ_em_ 408/678 nm) and tocopherols and phenols (λ_ex_/λ_em_ 293/322 nm) during the 21 months of storage. These bands decreased during the storage in the four VOOs due to the degradation reactions that were taking place [4].

The intensity of the band assigned to the pigments abruptly decreased during the first months of storage under moderate conditions (Figure 2a). Thus, this band completely disappeared in the fourth month of storage in VOO2 and VOO4, in the fifth month in VOO1, and in the eighteenth month in VOO3. The fluorescence intensity of this band for VOO3 was at least double compared to the other three oils before the storage (Figure 2a). This difference explained that this band required a longer time (18 months) to be undetected in the fluorescence spectra of VOO3. This high intensity matched with the high concentration of pheophytin a determined by HPLC in this oil: 23.43 mg/kg in VOO3, while the values for the rest of the oils were 7.06 in VOO1, 3.02 in VOO2, and 3.31 mg/kg in VOO4 (Table 2). Furthermore, the time-trend of the pheophytin a concentration determined by HPLC during the storage was similar to that of the fluorescence band assigned to pigments. Thus, the concentration values of pheophytin a also decreased in all of the cases during the storage (Table 2). These concentrations reached values close to zero (≤0.03 mg/kg) after 7 months of storage of the samples, except for VOO3, in which such a reduction was observed after 15 months (Table 2). The time-trend similarities between pheophytin a concentration (HPLC data) and the intensity of this band (λ_ex_/λ_em_ 408/678 nm) were supported by the high correlation coefficients between these two variables, which were 0.98 in VOO1, VOO2, and VOO3, and 0.79 in VOO4. The positive relationship between pheophytin a and the fluorescence band at λ_ex_/λ_em_ 408/678 nm was previously reported by several authors [2,4,29]. On the contrary, no relationship was found between the pyropheophytin a concentration and the fluorescence intensity of this band. In fact, the correlation coefficients in this case were 0.45 or lower in the four VOOs.

The intensity of the band assigned to tocopherols and phenols (λ_ex_/λ_em_ 293/322 nm) also decreased during storage. Unlike the band assigned to pigments, the intensity of this band never decreased to values close to zero, although it was reduced by approximately up to 50% of its initial values at the end of the experiment. However, as the band assigned to pigments, this band also underwent the highest decrease in the first five months of storage. Regarding the chemical analysis by HPLC, the concentrations of α-tocopherol and phenols also decreased during the storage experiment (Table 2). On the one hand, the α-tocopherol concentration underwent a reduction of their initial values of 51.84% for VOO1, 66.65% for VOO3, 67.87% for VOO2, and 89.44% for VOO4 at the end of the storage (Table 2). Nevertheless, they showed the highest decreases during the first five months of storage, which was also well represented by this fluorescence band. In fact, the correlation coefficients between HPLC results and the spectral intensity of this band in the whole storage experiment were 0.93 for VOO1 and VOO2, 0.94 for VOO3, and 0.79 for VOO4. On the other hand, the concentration of total phenols determined by HPLC revealed a decrease with respect to their initial concentration of 42.77% for VOO2, 54.40% for VOO4, 55.26% for VOO3, and 56.68% for VOO1 (Table 2). The correlation coefficients between the HPLC results and the intensity of this fluorescence band were 0.74 for VOO4, 0.79 for VOO1, 0.85 for VOO2, and 0.89 for VOO3. The individual contribution of tocopherols and phenols has been studied by synchronous fluorescence spectroscopy [4,24] and by using a vitamin E standard and a VOO phenol extract [48]. Some works used this band to develop models to estimate the concentration of tocopherols in vegetables oils [24,49] or even to classify oils according to their concentration of phenols [26].

In addition to the aforementioned bands, a new fluorescence band appeared at the intermediate-wavelength emission region (λ_ex_/λ_em_ 300–319/418 nm) during the storage. This band was previously reported and attributed to oil oxidation products by other authors [25,46,48,50]. Figure 2c shows the time-trend of the fluorescence intensity of this band in the VOOs during the storage time. In VOO4 and VOO2, this band was already observed at low intensity in the fresh oils, while in the rest of the VOOs it was barely detected. This agreed with the initial oxidation status of the samples according to the K_270_, K_232_, and peroxide value, which identified VOO2 and VOO4 as the most oxidized oils (Table 1). Furthermore, the fluorescence intensity of this band increased during the storage (Figure 2c), reaching its maximum in the last month of the storage. However, the time-trend of the fluorescence intensity of this band showed two different behaviors. Thus, VOO1 and VOO3 showed an abrupt increase of the fluorescence intensity of this band during the first five months, while it increased at a lower rate after this moment. VOO2 and VOO4, the two Arbequina oils, showed a different time-trend consisting in a continuous increment of the band intensity during the whole period and at lower rate compared to VOO1 and VOO3. During the storage experiment, all the quality parameters related to oxidation products (K_270_, K_232_, and peroxide value) also showed an increase (Table 1), which also reached their maximum value in the last month (twenty-first month) of storage. The highest relation of the intensity of this band with respect to the quality parameters previously mentioned was found for K_270_, which showed correlation coefficients of 0.70 for VOO2, 0.79 for VOO4, 0.85 for VOO1, and 0.92 for VOO3. The time-trend of K_270_ (Table 1) and the intensity of this fluorescence band (Figure 2c) showed that VOO1 and VOO3 were the most oxidized samples at the end of the experiment.

### 3.3. Multivariate Analysis of VOO Excitation–Emission Fluorescence Spectra

**Figure 3:**
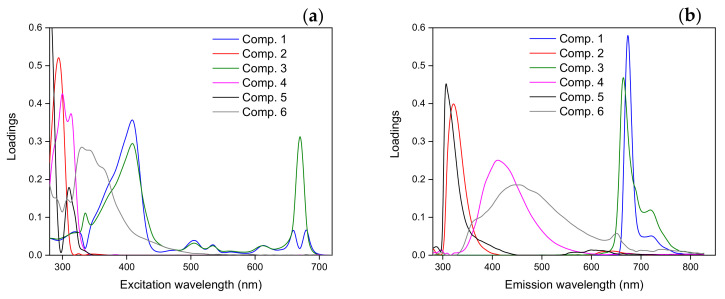
Parallel factor analysis (PARAFAC) excitation (**a**) and emission (**b**) profiles of the entire sample set (four monovarietal samples during the storage under moderate conditions) for the six components: Component 1 (λ_ex_/λ_em_ 408/678 nm), component 2 (λ_ex_/λ_em_ 293/322 nm), component 3 (λ_ex_/λ_em_ 408/668 nm), component 4 (λ_ex_/λ_em_ 300/418 nm), component 5 (λ_ex_/λ_em_ 280/314 nm), and component 6 (λ_ex_/λ_em_ 340/450 nm).

Multivariate exploratory methods were used to study the fluorescent compounds of the sample set. The 88 EEMs (22 EEMs per 4 VOOs, one per month during the 21 months of storage and the EEM of the fresh oil) were analyzed by the PARAFAC algorithm. The number of PARAFAC components was six, which was selected according to the core consistency (CONCORDIA = 87%) and the inspections of the residuals and the loadings (variance explained = 97.97%) [40,41]. Figure 3 shows the PARAFAC excitation and emission profiles for the 6 extracted components. The scores of PARAFAC components for each of the four studied oils are presented in Figure S4 (Supplementary Material). In order to carry out the analysis of the PARAFAC results, the six selected components were assigned to the fluorescent compounds. Firstly, the emission profiles of component 1 (λ_ex_/λ_em_ 408/678 nm) and component 3 (λ_ex_/λ_em_ 408/668 nm) were assigned to the chlorophyll pigments [2,4]. The existence of two components for chlorophyll pigments could be related with the fact that the emission wavelengths (maximum intensity) were different depending on the chlorophyll derivative and they varied in the range (658–672 nm) [51]. However, it was difficult to assign each component to one specific derivative. Secondly, the emission profiles of component 2 (λ_ex_/λ_em_ 293/322 nm) and component 5 (λ_ex_/λ_em_ 280/314 nm) were both assigned to tocopherols and phenols [21,26,29]. Previous research works dealing with the study of the individual contribution of tocopherols and phenols by means of synchronous fluorescence spectroscopy reported that tocopherols were related with higher excitation/emission wavelengths compared with phenols [21]. Therefore, it could be thought that component 2 would be more related with tocopherols and component 5 with phenols, although a mixture of contribution from both kinds of compounds was expected. Finally, the emission profiles of component 4 (λ_ex_/λ_em_ 300/418 nm) and component 6 (λ_ex_/λ_em_ 340/450 nm) were attributed to oxidized compounds by several studies [46,52,53,54].

**Figure 4:**
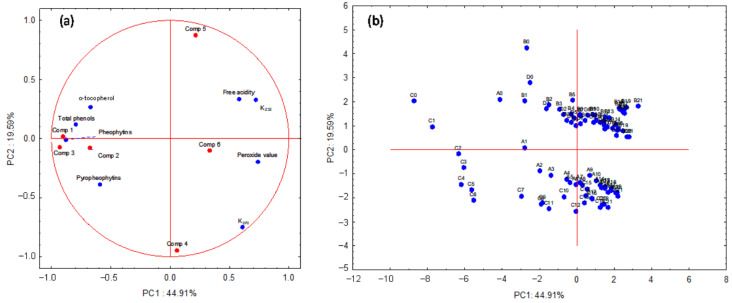
Principal component analysis (PCA) of all of physical–chemical parameters analyzed and the components extracted by PARAFAC analysis from the entire data set: Loading plot (**a**) and score plots (**b**) obtained of the two first principal components (PC1 and PC2). Codes: A, VOO1; B, VOO2; C, VOO3; D, VOO4. The numbers after the codes mean the months of the storage when the samples were collected.

A principal component analysis (PCA) was applied to the 6 PARAFAC components and the chemical and physical–chemical parameters (Table 1 and Table 2) to observe the distribution of the samples according to the storage time with a multivariate perspective. Figure 4 shows the loading (Figure 4a) and score (Figure 4b) plots obtained for the two first principal components (PC1 and PC2) of the PCA.

Figure 4a revealed that the physical-chemical parameters were distributed in two groups according to PC1. This distribution divided the parameters between those related to freshness markers (pheophytins and pyropheophytins) and antioxidant compounds (phenols and α-tocopherol), plotted in the negative side of PC1, and those related to oxidative and quality indexes of the oil (K_232_, K_270_, free acidity, and peroxide value) were placed in the positive side of PC1. The score plot presented in Figure 4b shows a sequential shift of the samples collected every month along the PC1 axis. The freshest oils were located in the left quadrant, which matched with the quadrant where pheophytins, pyropheophytins, phenols, and α-tocopherol were placed (Figure 4a). However, as storage progressed, the VOOs were plotted in the right quadrant, where the K_232_, K_270_, free acidity, and peroxide value were located. Due to the fact that the time is a continuous variable and the chemical changes between consecutive months are moderate in the collected samples, no clear groups were discriminated between samples. Nevertheless, the distribution of the stored oil samples along the PC2 (Figure 4b) was able to distinguish between Arbequina oils (VOO2 and VOO4) and the other two cultivars, Picual (VOO3) and Hojiblanca (VOO1).

The PCA was also used for studying the relationship between the physical–chemical parameters (peroxide value, K_232_, K_270_, free acidity, phenols, α-tocopherol, pheophytin, and pyropheophytin contents), which characterize the oils, and the fluorescence components obtained by PARAFAC. The PARAFAC components 1 and 3 (chlorophyll pigments), and 2 (tocopherols with contribution of phenols) were plotted near their related chemical parameters (pheophytins, pyropheophytins, phenols, and α-tocopherol) (Figure 4a). However, PARAFAC component 5, whose excitation and emission wavelengths are mainly assigned to phenols in the literature [21,26], was plotted far from this chemical parameter (Figure 4a). The high diversity of phenols and their different fluorescent characteristics [21,46] may partially explain the lack of correlation of component 5 with total phenol content. On the other hand, PARAFAC components 4 and 6 were located in the right quadrant, the same quadrant where the peroxide value, free acidity, K_232_, and K_270_ were plotted. The position of these two PARAFAC components in the loading plot (Figure 4a) indicated that they were related to oxidation products. The relation of the emission region (400–600 nm) of the components 4 and 6 with the oxidation products were reported in previous studies [4,50,53].

**Figure 5:**
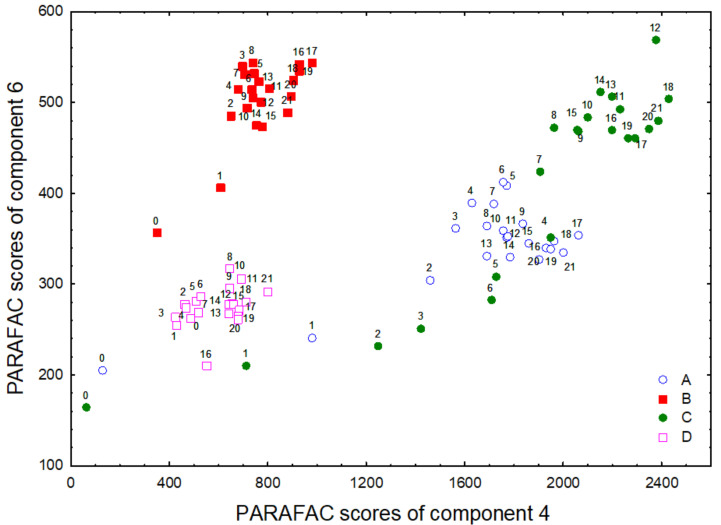
2D plot of components 4 (λ_ex_/λ_em_ 300/418 nm) and 6 (λ_ex_/λ_em_ 340/450 nm) for all VOOs during the entire storage time. Codes: A, VOO1 (blue circle); B, VOO2 (red square); C, VOO3 (green circle); D, VOO4 (pink square). The numbers mean the months of the storage when the samples were collected.

PARAFAC components 4 (λ_ex_/λ_em_ 300/418 nm) and 6 (λ_ex_/λ_em_ 340/450 nm) were represented in a 2D plot, which is shown in Figure 5, to analyze the differences between the four VOOs according to their oxidation state in the course of the storage. The intensity of both components revealed a different oxidation state of the VOOs at the beginning of the storage. Despite the intensity of both components changing over time, the differences of the oxidation state between oils were maintained according to these two components. Thus, four distinguishable groups were observed in the 2D plot associated with the four VOOs. The most remarkable increase of the component intensities was observed in the first months of storage (approximately 0–5 months) (Figure 5). The correlation study of components 4 and 6 with respect to the oxidation indexes (K_232_, K_270_, peroxide value) revealed that the best correlation coefficients were found for component 4 and K_270_ (R = 0.89, 0.80, 0.93, and 0.81 for oils VOO1, VOO2, VOO3, and VOO4, respectively). 

A further study was carried out with a stepwise linear discriminant analysis (SLDA) using all PARAFAC components in order to select which one was the most efficient at discriminating between fresh and aged oils. For this aim, only the samples at the beginning (0–5 months) and at the end (16–21 months) of the experiment were considered as the two classes to be distinguished in the classification model. The selection of the classifying variables (PARAFAC components) included in the model was carried out through F-to-enter and F-to-remove values [55]. The procedure selected components 2 and 4 from the initial six PARAFAC components to build the classification model. These two variables, associated with tocopherols with the contribution of phenols (component 2) and oxidation products (component 4), provided complementary information about the chemical changes that are taking place during storage.

### 3.4. Sensory Quality Changes in the Samples Analyzed by Fluorescence Spectroscopy

The sensory assessment of the fresh oils determined that all VOOs were within “extra virgin olive oil” category according to European regulation [15], except VOO4. This oil was categorized within the “virgin olive oil” category, due to a winey-vinegary defect (Md = 2.1) detected by the panelists before starting the storage. The panelists classified the fresh oils according to their medians of the fruity attribute in the following order: VOO1 (Mf = 4.7) > VOO3 (Mf = 3.8) > VOO2 (Mf = 3.5) > VOO4 (Mf = 3.0).

During storage under moderate conditions, the panelists detected some changes in the flavor of the oils. As it was highlighted in a previous publication, these changes are explained by the changes in the volatile composition during the storage [13]. These changes were enough to lead a change in their categories at different time during the storage. Table S1 (Supplementary Material) shows the changes produced in the median of the defect and fruity attribute during the storage experiment. The median of the fruity attribute decreased in the four oils during the storage experiment. VOO2 showed the fastest decrease, so it was reduced by 57.15% of its initial value during the first five months of storage. Furthermore, the increment of its median of the defect (Md = 1.0) in the fifth month of storage resulted in a downgrading of category to “virgin olive oil”, due to a detection of a winey-vinegary defect at this time. VOO3 changed to the “virgin olive oil” category in the mid-term of the storage (tenth storage month) when a winey-vinegary defect (Md = 2.6) was detected by the panelists. VOO1 was the oil that remained unchanged in its category longer, changing to “virgin olive oil” category in the fifteenth month of storage, because an incipient rancid defect (Md = 2.5) was detected. VOO4 was the only oil that reached the “lampante virgin olive oil” category during the storage; the median of the defect reached a value of 3.5 in the eighteenth month.

The study of the physical–chemical parameters during the VOO storage revealed that the category downgrading was due to the increment of K_270_ and the changes in the median of fruity and defect values (Table 1 and Table S1). However, the downgrading of category according to K_270_ occurred in the first 4 months of storage, while the detection of sensory defects occurred at different moments (between 5 and 18 months) depending on the oil. This fact revealed the complexity of quality changes during the storage in which each physical–chemical parameter informed a different aspect of quality. Any analytical method being proposed to control virgin olive oil degradation should consider this complexity. In particular, sensory quality needs special attention due to the discrepancies sometimes found in the sensory assessment results, which is considered by the regulatory bodies as a main problem in the quality assessment of VOO. Furthermore, the sensory quality is the characteristic most appreciated by consumers; therefore, its control during the commercialization of VOO should be extremely important. Although fluorescence spectroscopy determined compounds that were not related to sensory defects, it is important to know if the changes determined by this method occurred before or after sensory defects were clearly detected by panelists and consequently a category downgrading took place. Thus, this information is necessary for a correct interpretation of the results. 

**Figure 6:**
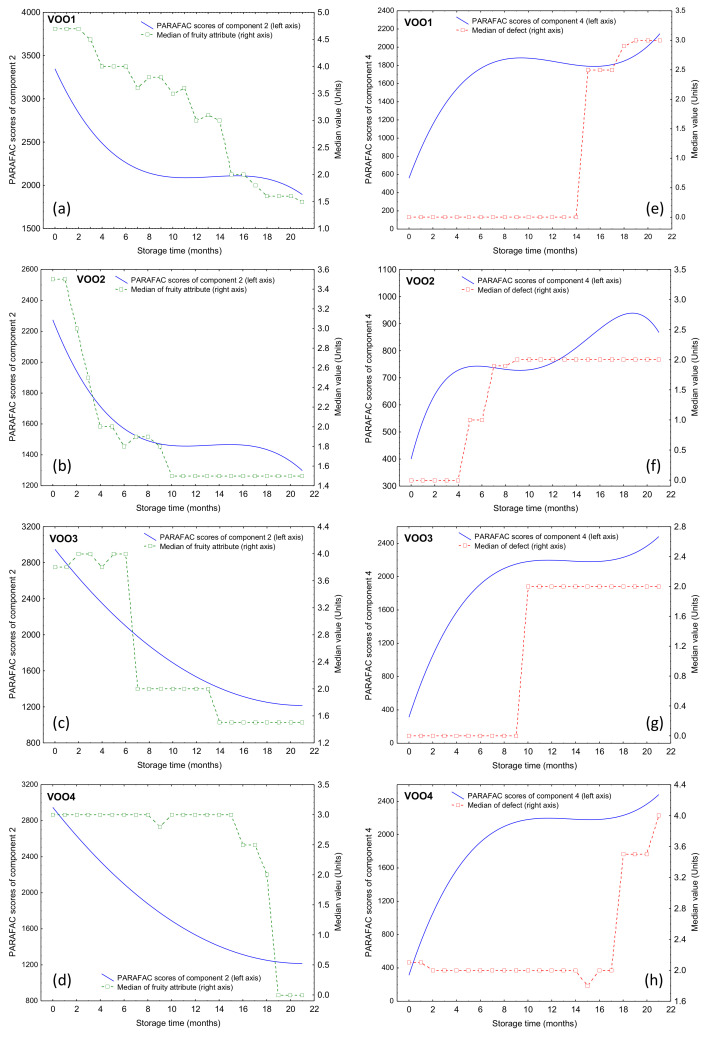
Time-trend of the median of fruity and defect values during the storage per each VOO, which is represented together with the time-trend of the PARAFAC components 2 (**a**–**d**) and 4 (**e**–**h**) in a double-y graph, respectively.

Figure 6 shows the median values of the defect and fruity attribute during the storage, together with the time-trend of the PARAFAC components 2 and 4, which were the components previously selected by SLDA. The median of the fruity attribute decreased at the same rate as component 2 (associated with tocopherols and phenols), the correlation coefficients between both variables being 0.70, 0.88, 0.90, 0.52 for VOO1, VOO2, VOO3, and VOO4, respectively. Only the latter showed a correlation coefficient lower than 0.70, probably due to the fact that this oil was already within the “virgin olive oil” category and its median of fruity attribute was the lowest among the studied oils (Mf = 3.0).

In the case of the median of the defect, this variable was related to component 4, associated with oxidation products. In this case, the changes in the median of the defect were marked, unlike the median of fruity attribute, increasing from zero to approximately 2, while the change of component 4 intensity was continuous during the storage. It was also important to note that the increase of the median of the defect could be due to the detection of rancidity, associated with oxidation products, but also to the detection of some fermentative defects, such as the winey-vinegary defect, already existing in the fresh samples and masked by the fruitiness. That explains the low correlation coefficients between the median of the defect and component 4 for the four oils (0.46, 0.67, 0.66, and 0.34 for VOO1, VOO2, VOO3, and VOO4, respectively). In these oils, the increase of the intensity of component 4 up to the plateau was observed before the abrupt change in the median of the defect.

## 4. Conclusions

This study verified the ability of fluorescence spectroscopy to monitor the chemical changes of virgin olive oils during storage under moderate conditions and assessed the relationship with the different quality parameters. In this study, 4 monovarietal VOOs were examined in their stability. The samples were obtained by different producers. The quality and the stability of the samples were influenced by many variables (the state of olive ripeness, the method of extraction, the geographical provenance, and the agricultural practices, among many other factors). This explains that the varietal influence was not so evident for some parameters. For example, the two Arbequina oils presented different phenol concentrations and changes in the sensory characteristics. 

The main correlations between the bands identified in the excitation–emission matrices and the quality parameters were found in: The intensity of the band assigned to pigments (λ_ex_/λ_em_ 408/678 nm) with the concentration of pheophytin a, the intensity of the band assigned to tocopherols and phenols (λ_ex_/λ_em_ 293/322 nm) with the concentration of α-tocopherol, and the intensity of the band assigned to oxidation products (λ_ex_/λ_em_ 300/418 nm) with K_270_. Excitation–emission fluorescence spectroscopy combined with PARAFAC analysis could give information about fluorescent compounds that contribute to the fluorescence emission of VOO, thereby providing a degradation map of the oil. The components extracted by PARAFAC were associated with certain groups of compounds and therefore the observed changes could be interpreted according to the related quality parameters determined in the same oils. Thus, a study of all this information permitted a correct interpretation of the spectra. PARAFAC components 2 and 4 were selected as the best components to distinguish between fresh and aged oils. Both components provided complementary information since they informed on the content of tocopherols with contribution of phenols (component 2) and oxidation products (component 4). Due to the importance of the VOO sensory characteristics for consumer acceptation, and considering that one of the main reasons for downgrading the oils to a lower quality category is the detection of sensory defects in aged oils, the sensory evaluation of the samples was also studied in relation to components 2 and 4 in order to have information of all kinds of degradations. In both components, the changes in their intensity were observed at the same time, or even earlier, than the changes in the medians of the fruity attribute and the defect were determined. This observation could be used as a basis for future studies centered on the interpretation of fluorescent spectra for a practical application in aging control of oils. The challenges ahead should be focused on establishing rules for an easy interpretation of the fluorescence spectra by producers for a daily routine analysis, and also verifying if these rules are dependent on the cultivars. Fluorescence spectroscopy is still scarcely distributed in the labs of olive oil companies, although this technique is affordable and it does not require special training.

## Figures and Tables

**Table 1:** Quality parameters (peroxide value, free acidity or FFA, K_270_ and K_232_) are shown for the four fresh virgin olive oils (VOOs) before starting the storage (“time zero”) and in every month during the entire storage time. According to European commission regulation [15]: Limits for extra virgin olive oil: Peroxide value ≤ 20 meq O_2_/kg, FFA ≤ 0.8%, K_270_ ≤ 0.22, K_232_ ≤ 2.50. Limits for virgin olive oil: Peroxide value ≤ 20 meq O_2_/kg, FFA ≤ 2.0%, K_270_ ≤ 0.25, K_232_ ≤ 2.60.

	Peroxide Value (meq O_2_/kg)				FFA (% m/m Oleic Acid)				K_270_ (Absorbance Units)				K_232_ (Absorbance Units)			
Months of Storage	VOO1	VOO2	VOO3	VOO4	VOO1	VOO2	VOO3	VOO4	VOO1	VOO2	VOO3	VOO4	VOO1	VOO2	VOO3	VOO4
0	4.30	5.13	3.63	4.82	0.15	0.21	0.20	0.20	0.06	0.18	0.04	0.18	1.53	1.87	1.73	1.84
1	7.54	5.37	4.12	4.94	0.16	0.22	0.21	0.22	0.21	0.18	0.17	0.20	1.95	1.87	1.78	1.96
2	7.77	5.17	4.85	4.74	0.16	0.21	0.21	0.22	0.24	0.19	0.18	0.21	1.93	1.83	1.82	1.97
3	7.56	5.27	5.45	5.30	0.18	0.22	0.21	0.21	0.23	0.20	0.22	0.22	1.88	1.82	1.82	1.96
4	7.46	5.86	5.48	6.22	0.18	0.22	0.21	0.21	0.25	0.22	0.22	0.21	1.92	1.89	1.82	1.95
5	7.69	5.98	5.63	5.83	0.19	0.23	0.21	0.21	0.26	0.23	0.22	0.23	1.90	1.85	1.77	1.95
6	7.63	5.77	6.18	6.17	0.19	0.23	0.21	0.21	0.27	0.23	0.24	0.23	1.91	1.97	1.80	1.96
7	8.31	5.68	5.93	6.11	0.19	0.23	0.22	0.22	0.27	0.23	0.25	0.23	1.91	1.90	1.80	1.97
8	8.38	5.98	5.99	7.11	0.20	0.24	0.22	0.23	0.27	0.24	0.28	0.24	1.93	1.90	1.82	1.98
9	9.19	7.30	6.24	8.12	0.21	0.26	0.23	0.24	0.28	0.24	0.27	0.23	1.97	1.90	1.84	1.98
10	10.04	7.48	6.74	9.28	0.21	0.27	0.23	0.23	0.29	0.24	0.28	0.24	1.97	1.88	1.83	1.98
11	10.60	8.36	6.66	9.73	0.22	0.28	0.22	0.24	0.30	0.26	0.29	0.24	1.97	1.92	1.83	1.99
12	10.69	8.39	7.27	10.31	0.21	0.27	0.23	0.24	0.32	0.26	0.33	0.24	1.94	1.93	1.84	1.99
13	11.64	8.86	7.72	10.56	0.22	0.28	0.22	0.25	0.31	0.26	0.31	0.25	1.97	1.97	1.84	2.00
14	11.80	9.47	7.45	10.33	0.21	0.28	0.23	0.25	0.31	0.26	0.28	0.25	1.97	1.99	1.85	2.03
15	11.25	9.32	7.32	10.70	0.22	0.29	0.23	0.26	0.32	0.26	0.30	0.26	1.98	2.12	1.85	2.04
16	11.94	9.43	7.41	10.74	0.21	0.29	0.24	0.27	0.32	0.26	0.30	0.26	2.02	2.05	1.86	2.07
17	11.97	9.78	7.60	10.61	0.22	0.30	0.23	0.27	0.31	0.26	0.31	0.26	1.99	2.06	1.85	2.07
18	11.98	9.82	8.01	10.88	0.23	0.29	0.23	0.27	0.32	0.26	0.33	0.27	2.04	2.06	1.90	2.07
19	12.54	10.48	8.05	11.06	0.23	0.30	0.23	0.27	0.33	0.26	0.32	0.27	1.99	2.07	1.89	2.07
20	13.06	10.33	8.89	11.75	0.22	0.31	0.23	0.28	0.33	0.26	0.32	0.27	1.98	2.02	1.90	2.07
21	13.59	10.64	8.84	12.38	0.23	0.30	0.23	0.29	0.33	0.28	0.33	0.28	2.04	2.23	1.90	2.08

**Table 2:** Concentrations of phenols, α-tocopherol, and pigments derived from chlorophyll a (pheophytin a and pyropheophytin a) are shown for the four fresh virgin olive oils (VOOs) before starting the storage (“time zero”) and in every month during the entire storage time.

	Total Phenols (mg/kg)				α-Tocopherol (mg/kg)				Pheophytin a (mg/kg)				Pyropheophytin a (mg/kg)			
Months of Storage	VOO1	VOO2	VOO3	VOO4	VOO1	VOO2	VOO3	VOO4	VOO1	VOO2	VOO3	VOO4	VOO1	VOO2	VOO3	VOO4
0	246.71	338.90	564.82	451.25	212.62	272.28	256.91	192.94	7.06	3.02	23.43	4.61	0.03	0.04	0.07	0.11
1	239.51	337.08	558.45	422.98	190.47	260.76	210.03	157.49	7.68	3.00	22.98	4.60	0.14	0.04	0.39	0.26
2	238.82	338.66	547.93	399.56	160.05	220.01	134.21	125.84	5.17	1.30	22.59	0.53	0.33	0.05	0.63	0.12
3	231.70	333.44	521.74	381.09	147.63	194.34	134.78	122.78	2.60	0.43	21.32	0.33	0.37	0.05	0.95	0.16
4	218.11	325.57	499.02	373.03	140.11	187.77	140.81	120.65	0.49	0.12	16.73	0.20	0.22	0.04	1.18	0.09
5	209.46	316.30	486.27	360.98	136.22	178.83	138.86	115.21	0.47	0.11	16.83	0.19	0.20	0.04	2.74	0.07
6	204.68	308.34	474.05	348.81	133.77	162.18	131.86	115.52	0.18	0.10	17.17	0.12	0.08	0.03	2.13	0.06
7	198.97	298.84	459.09	340.11	130.48	154.93	137.04	110.22	0.03	0.03	5.36	0.03	0.03	0.02	1.23	0.02
8	190.74	292.30	441.80	324.71	131.55	147.99	129.99	92.26	0.03	0.05	2.22	0.03	0.05	0.02	1.22	0.02
9	183.06	280.30	417.35	315.62	129.12	136.40	129.09	85.48	0.02	0.04	2.39	0.05	0.03	0.02	1.72	0.02
10	168.05	271.11	405.58	312.19	125.98	123.43	124.91	81.60	0.03	0.02	0.44	0.03	0.02	0.02	1.58	0.01
11	155.06	261.88	392.51	287.31	122.78	113.15	116.48	79.05	0.02	0.04	0.62	0.02	0.04	0.02	1.51	0.02
12	149.66	253.01	366.32	273.11	122.30	112.77	111.11	75.12	0.02	0.03	0.27	0.02	0.02	0.02	0.54	0.02
13	144.38	247.86	351.22	260.54	121.81	109.56	93.18	73.43	0.02	0.04	0.13	0.02	0.02	0.01	0.32	0.02
14	138.29	240.03	339.06	253.55	122.06	107.97	92.08	70.76	0.01	nd	0.09	nd	0.02	0.02	0.22	nd
15	131.43	231.23	325.29	235.88	120.36	103.17	90.44	60.51	nd	nd	nd	nd	nd	0.03	nd	nd
16	128.41	222.52	326.64	225.60	119.64	99.73	92.84	54.64	nd	nd	nd	nd	nd	0.03	nd	nd
17	124.19	217.34	314.18	225.25	114.55	97.04	93.07	59.10	nd	nd	nd	nd	nd	0.03	nd	nd
18	118.84	209.22	290.96	223.78	105.07	96.47	91.47	53.67	nd	nd	nd	nd	nd	0.03	nd	nd
19	112.45	203.57	277.17	205.86	106.30	96.94	84.28	50.20	nd	nd	nd	nd	nd	0.03	nd	nd
20	108.06	199.34	265.78	205.83	102.30	91.28	84.92	22.42	nd	nd	nd	nd	nd	0.03	nd	nd
21	106.87	193.95	252.69	205.76	102.39	87.47	85.69	20.38	nd	nd	nd	nd	nd	0.03	nd	nd

Note: nd; not detected.

## Supplementary Materials

The following are available online at https://www.mdpi.com/2304-8158/9/12/1846/s1, Table S1: Sensory assessment results (medians of the fruity attribute and defect) during the storage experiment for each VOOs. Table S2: Phenolic compounds identified in the VOO samples subjected to storage under moderate conditions. The compounds are grouped according to the excitation wavelength chromatogram where they were registered. Figure S1: An example of chromatogram obtained from the phenol analysis. The chromatograms correspond to VOO1 (fresh oil). The codes are shown in Table S3. Figure S2: An example of chromatogram obtained from the α-tocopherol analysis. The chromatogram corresponds to VOO1 (fresh oil). Figure S3: An example of chromatogram of the degradation products of chlorophyll a obtained in the analysis. The chromatogram corresponds to VOO3 (fresh oil). Figure S4: PARAFAC scores of the sample set (different monovarietal samples during the storage under moderate conditions). Component 1 (λ_ex_/λ_em_ 408/678 nm), component 2 (λ_ex_/λ_em_ 293/322 nm), component 3 (λ_ex_/λ_em_ 408/668 nm), component 4 (λ_ex_/λ_em_ 300/418 nm), component 5 (λ_ex_/λ_em_ 280/314 nm), and component 6 (λ_ex_/λ_em_ 340/450 nm).
