# Supplementary material for: Monitoring Virgin Olive Oil Shelf-Life by Fluorescence Spectroscopy and Sensory Characteristics: A Multidimensional Study Carried Out under Simulated Market Conditions
Source: Foods. 2020 Dec 11;9(12):1846. doi: 10.3390/foods9121846 (PMC7763516; doi:10.3390/foods9121846)
Supplement: Supplementary file 1 [file foods-09-01846-s001.pdf]

*Supplementary Material*

# Monitoring virgin olive oil shelf-life by fluorescence spectroscopy and sensory characteristics. A multidimensional study carried out under simulated market conditions.

**Ana Lobo-Prieto[a], Noelia Tena[b], Ramon Aparicio-Ruiz[b], Diego L. García-González[a]*, Ewa Sikorska[c].**

[a]Instituto de la Grasa (CSIC), Campus Universidad Pablo de Olavide – Edificio 46, Ctra. de Utrera, Km. 1, 41013 Sevilla, Spain.

[b]Department of Analytical Chemistry, Universidad de Sevilla, Prof. García González 2, 41012 Sevilla, Spain.

[c]Institute of Quality Science, The Poznan University of Economics and Business, al. Niepodleglosci 10, 61-875 Poznań, Poland.

**Table S1.** Sensory assessment results (medians of the fruity attribute and defect) during the storage experiment for each VOO.

| **Months of storage** | **Median of the fruity attribute ($M_f$)** | | | | **Median of defect ($M_d$)** | | | |
|---|---|---|---|---|---|---|---|---|
| | **VOO1** | **VOO2** | **VOO3** | **VOO4** | **VOO1** | **VOO2** | **VOO3** | **VOO4** |
| 0 | 4.7 | 3.5 | 3.8 | 3.0 | 0.0 | 0.0 | 0.0 | 2.0 |
| 1 | 4.7 | 3.5 | 3.8 | 3.0 | 0.0 | 0.0 | 0.0 | 2.0 |
| 2 | 4.7 | 3.0 | 3.8 | 3.0 | 0.0 | 0.0 | 0.0 | 2.0 |
| 3 | 4.5 | 2.5 | 3.8 | 3.0 | 0.0 | 0.0 | 0.0 | 2.0 |
| 4 | 4.0 | 2.0 | 3.8 | 3.0 | 0.0 | 0.0 | 0.0 | 2.0 |
| 5 | 4.0 | 2.0 | 3.8 | 3.0 | 0.0 | 1.0 | 0.0 | 2.0 |
| 6 | 4.0 | 1.9 | 3.8 | 3.0 | 0.0 | 1.0 | 0.0 | 2.0 |
| 7 | 3.8 | 1.9 | 2.0 | 3.0 | 0.0 | 1.9 | 0.0 | 2.0 |
| 8 | 3.8 | 1.9 | 2.0 | 3.0 | 0.0 | 1.9 | 0.0 | 2.0 |
| 9 | 3.8 | 1.8 | 2.0 | 2.8 | 0.0 | 1.9 | 2.6 | 2.0 |
| 10 | 3.6 | 1.5 | 2.0 | 2.8 | 0.0 | 2.0 | 2.5 | 2.0 |
| 11 | 3.6 | 1.5 | 2.0 | 2.8 | 0.0 | 2.0 | 2.5 | 2.0 |
| 12 | 3.1 | 1.5 | 2.0 | 2.8 | 0.0 | 2.0 | 2.5 | 2.0 |
| 13 | 3.1 | 1.5 | 2.0 | 2.8 | 0.0 | 2.0 | 2.5 | 2.0 |
| 14 | 3.0 | 1.5 | 1.8 | 2.8 | 0.0 | 2.0 | 2.0 | 2.0 |
| 15 | 2.0 | 1.5 | 1.8 | 2.8 | 2.5 | 2.0 | 2.0 | 2.0 |
| 16 | 2.0 | 1.5 | 1.8 | 2.5 | 2.5 | 2.0 | 2.0 | 2.0 |
| 17 | 1.8 | 1.5 | 1.8 | 2.5 | 2.5 | 2.0 | 2.0 | 2.0 |
| 18 | 1.6 | 1.5 | 1.8 | 2.0 | 2.9 | 2.0 | 2.0 | 3.5 |
| 19 | 1.6 | 1.5 | 1.8 | 0.0 | 3.0 | 2.0 | 2.0 | 3.5 |
| 20 | 1.6 | 1.5 | 1.8 | 0.0 | 3.0 | 2.0 | 2.0 | 3.5 |
| 21 | 1.5 | 1.5 | 1.8 | 0.0 | 3.0 | 2.0 | 2.0 | 4.0 |

*Note: According to European commission regulation (see* [15] *in the main publication): Extra virgin olive oil, $M_f$ >0 and $M_d$ =0; Virgin olive oil, $M_f$ >0 and $M_d$ ≤3.5; Lampante olive oil, $M_d$ >3.5.*

**Table S2.** Phenolic compounds identified in the VOO samples subjected to storage at moderate conditions. The compounds are grouped according to the excitation wavelength chromatogram where they were registered.

| **Compound** | **Code** |
|---|---|
| Identified at $\lambda_{ex}$ = 280 nm | |
| Hydroxytyrosol | 1 |
| Tyrosol | 2 |
| *p*-hydroxyphenylacetic acid (internal standard) | 3 |
| Vanillic acid | 4 |
| Vanillin | 5 |
| *p*-coumaric acid | 6 |
| Hydroxytyrosol acetate | 7 |
| *o*-coumaric acid (internal standard) | 11 |
| Dialdehydic form of elenolic acid linked to hydroxytyrosol (3,4-DHPEA-EDA) | 12 |
| Dialdehydic form of decarboxymethyl elenolic acid liked to *p*-HPEA (*p*-HPEA-EDA) | 14 |
| Pinoresinol | 15 |
| Cinamic acid | 16 |
| Acetoxypinoresinol | 17 |
| Aldehydic form of elenolic acid linked to hydroxytyrosol (3,4-DHPEA-EA) | 20 |
| Aldehydic form of elenolic acid linked to tyrosol (*p*-HPEA-EA) | 22 |
| Identified at $\lambda_{ex}$ = 235 nm | |
| Elenoic acid | A |
| Identified at $\lambda_{ex}$ = 335 nm | |
| Luteolin | 19 |
| Apigenin | 21 |

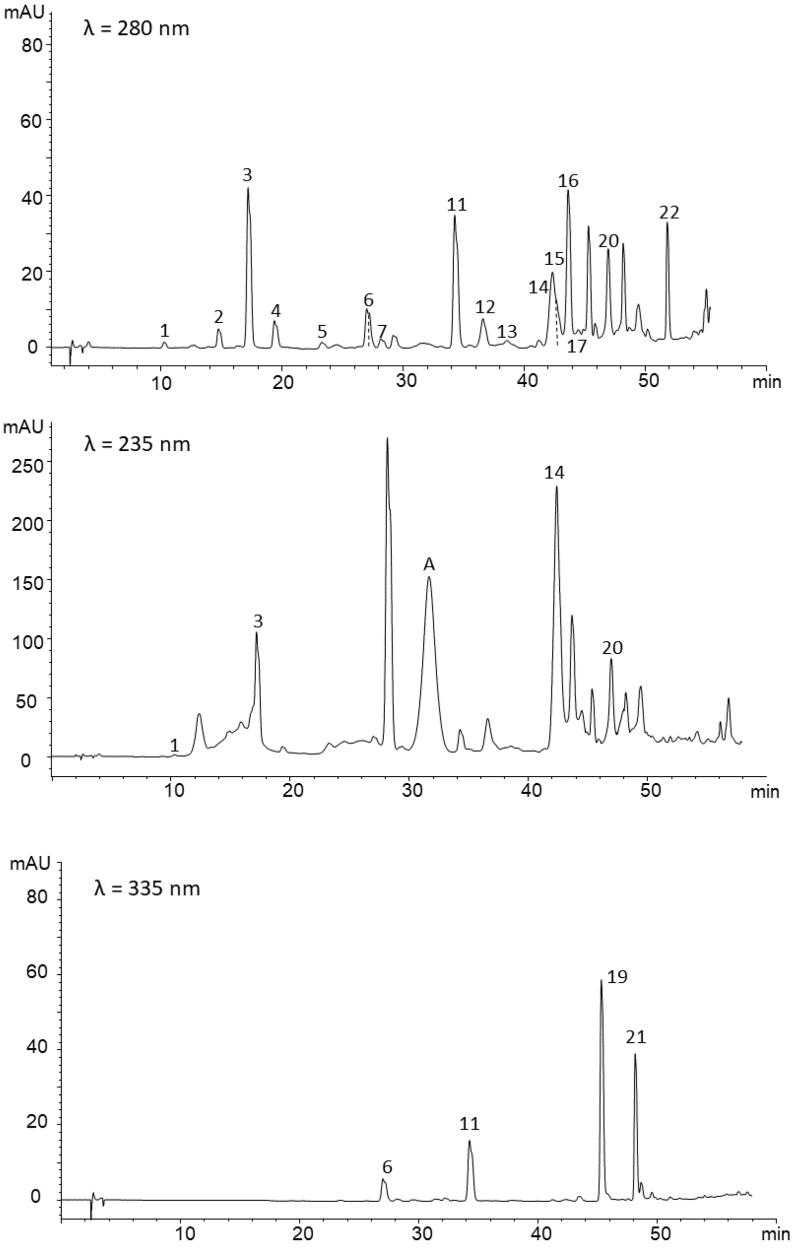


**Figure S1.** An example of chromatogram obtained from the phenol analysis. The chromatograms correspond to VOO1 (fresh oil). The codes are shown in **Table S2**.

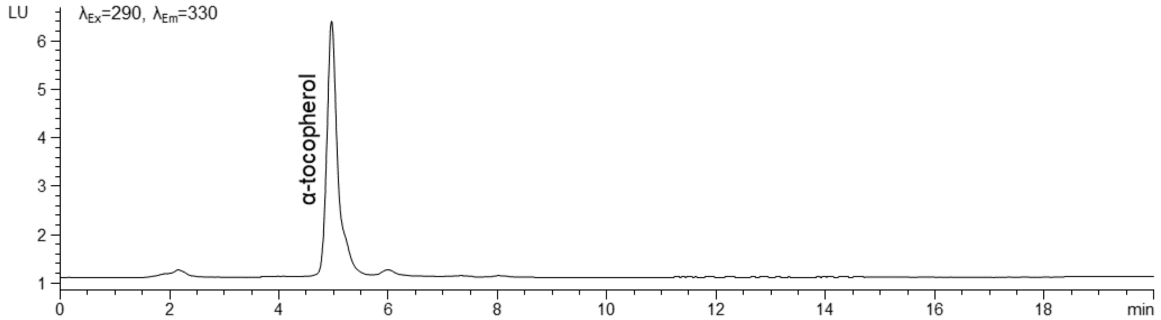


**Figure S2.** An example of chromatogram obtained from the α-tocopherol analysis. The chromatogram corresponds to VOO1 (fresh oil).

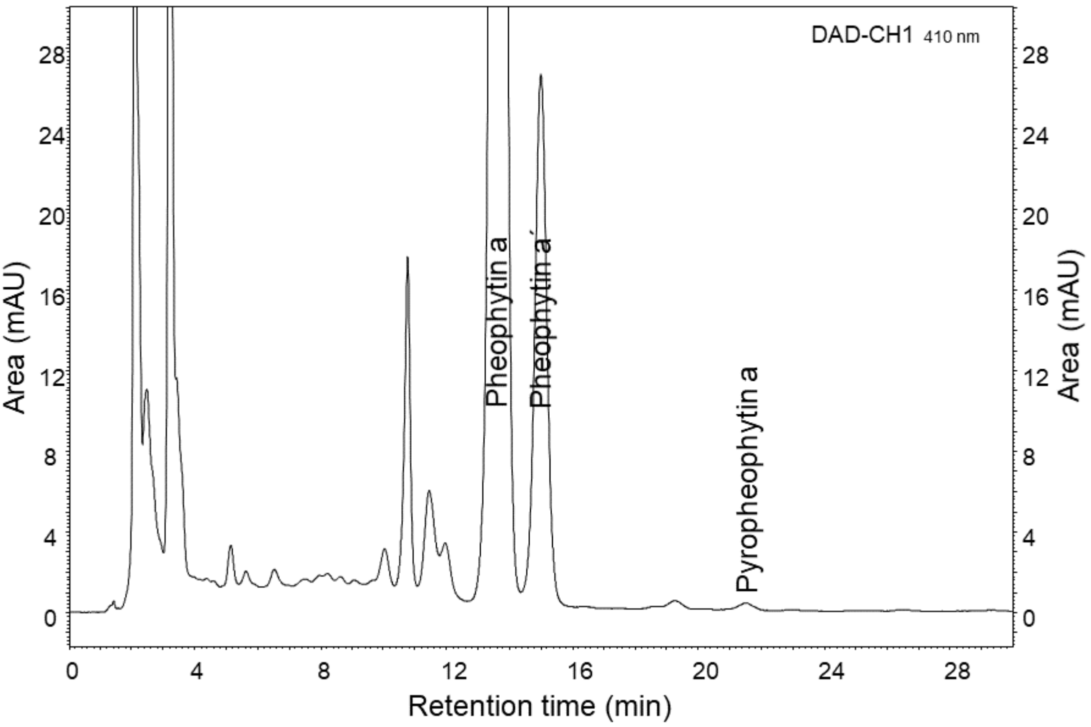


**Figure S3.** An example of chromatogram of the degradation products of chlorophyll a obtained in the analysis. The chromatogram corresponds to VOO3 (fresh oil).

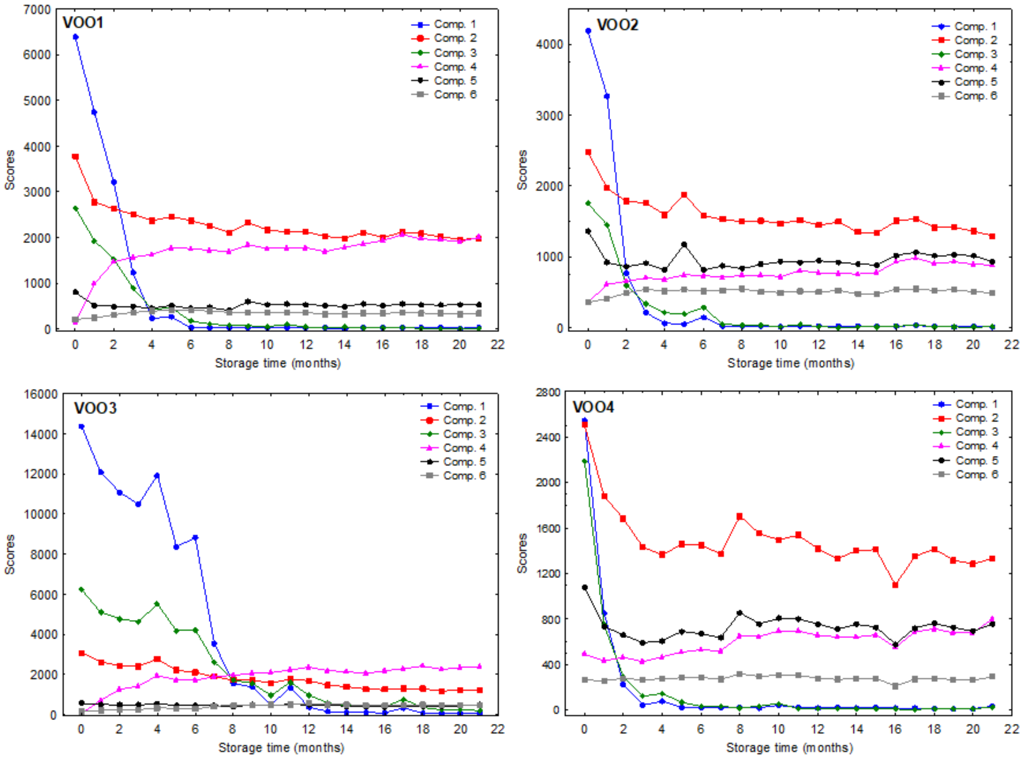


**Figure S4.** PARAFAC scores of the sample set (different monovarietal samples during the storage at moderate conditions). Component 1 ($\lambda_{ex}/\lambda_{em}$ 408/678 nm), component 2 ($\lambda_{ex}/\lambda_{em}$ 293/322 nm), component 3 ($\lambda_{ex}/\lambda_{em}$ 408/668 nm), component 4 ($\lambda_{ex}/\lambda_{em}$ 300/418 nm), component 5 ($\lambda_{ex}/\lambda_{em}$ 280/314 nm), and component 6 ($\lambda_{ex}/\lambda_{em}$ 340/450 nm).
